# Geptop: A Gene Essentiality Prediction Tool for Sequenced Bacterial Genomes Based on Orthology and Phylogeny

**DOI:** 10.1371/journal.pone.0072343

**Published:** 2013-08-15

**Authors:** Wen Wei, Lu-Wen Ning, Yuan-Nong Ye, Feng-Biao Guo

**Affiliations:** Center of Bioinformatics and Key Laboratory for NeuroInformation of Ministry of Education, School of Life Science and Technology, University of Electronic Science and Technology of China, Chengdu, China; Université Paris-Sud, France

## Abstract

Integrative genomics predictors, which score highly in predicting bacterial essential genes, would be unfeasible in most species because the data sources are limited. We developed a universal approach and tool designated Geptop, based on orthology and phylogeny, to offer gene essentiality annotations. In a series of tests, our Geptop method yielded higher area under curve (AUC) scores in the receiver operating curves than the integrative approaches. In the ten-fold cross-validations among randomly upset samples, Geptop yielded an AUC of 0.918, and in the cross-organism predictions for 19 organisms Geptop yielded AUC scores between 0.569 and 0.959. A test applied to the very recently determined essential gene dataset from the *Porphyromonas gingivalis*, which belongs to a phylum different with all of the above 19 bacterial genomes, gave an AUC of 0.77. Therefore, Geptop can be applied to any bacterial species whose genome has been sequenced. Compared with the essential genes uniquely identified by the lethal screening, the essential genes predicted only by Gepop are associated with more protein-protein interactions, especially in the three bacteria with lower AUC scores (<0.7). This may further illustrate the reliability and feasibility of our method in some sense. The web server and standalone version of Geptop are available at http://cefg.uestc.edu.cn/geptop/ free of charge. The tool has been run on 968 bacterial genomes and the results are accessible at the website.

## Introduction

Essential genes are the genes which are “essential” for survival of an organism [1], [2]. Therefore, identification of gene essentiality is important in understanding the minimal requirements for cell survival and functionality [3], [4]. The study of essential genes is an important step towards understanding the evolution of microbes [5]. Systematic genome-wide interrogations, such as single-gene knockouts [6], [7], transposon mutagenesis [8], [9], [10] and RNA interference [11], [12], have been used to identify essential genes. However, such experiential techniques are challenging and time-consuming. High-efficiency computational methods offer an appealing alternative for predicting essential genes without the expense and difficulty of an experimental screen.

An initial computational approach, by comparing the genomes of *Haemophilus influenzae* and *Mycoplasma genitalium*, identified approximately 250 candidate essential genes as the minimal gene set [3]. These genes were considered necessary for the survival of *H. influenzae* and *M. genitalium*. Bacterial essential gene products are often attractive drug targets in the development of antibiotics. Several previous studies relied upon homology mapping against an experimentally determined set of essential genes to identify drug targets [13], [14], [15], [16], [17], [18], [19]. The evolutionary distance between genomes can have a significant impact on the outcome of comparative genomic analyses [20]. On the other hand, orthologs of essential genes do not always carry out essential functions among those closely related organisms, and may even be absent in certain situations [21]. When an essential gene is lost, it is possible for a living cell to be rescued through the over-expression of a non-homologous and non-essential gene [5]. Consequently, the number of genes identified in the minimal set using comparative genomics across many bacterial species decreased significantly from comparing of *H. influenzae* and *M. genitalium* [20], [22].

Flux balance analysis is a constraint-based modeling technique used to simulate fluxes of metabolic networks at the steady-state. It can be used to identify minimal gene requirements. The essential genes in several bacteria have been predicted based on this method [23], [24], [25]. An accuracy of 85% was obtained in predicting yeast essential genes [26]. Flux balance analysis is a powerful approach for predicting essential genes. However, it is strongly dependent on the knowledge of metabolic networks.

It has been found that several classes of biological features are correlated with gene essentiality and they are used to predict essential genes. As an example, a gene with an essential function is likely to use optimal codons, to be located on the leading strand, and to be with a high centrality [27], [28], [29], [30], [31]. In general, genomic features fall into three categories: intrinsic features based on sequences, those derived from sequences, and data from functional genomics experiments. Machine learning systems based on integrative features have been trained to identify essential genes in *Saccharomyces cerevisiae* [32], [33], [34]. Chen et al. investaged the relationship between the gene fitness of *S. cerevisiae* gene and the features derived from the high-throughput data [32]. They selected the fitness associated factors to predict the fitness of individual protein by machine learning methods. Seringhaus et al. [34] used only sequence dependent features to estimate essentiality for yeast proteins. The study showed excellent performance: a ten-fold cross-validation in yeast with a probability threshold of 0.5 correctly classified over 80% of a total of 4648 genes. The organism-wise cross-validation between *Escherichia coli* and *Pseudomonas aeruginosa* yielded the area under curve (AUC) scores of 0.75–0.81 in the receiver operating curves (ROC) using 33 broad variables [35]. Cross-organism prediction on four bacteria yielded AUC scores between 0.69 and 0.89, based on 13 integrative biological features [36]. Of the 13 features, the protein domain enrichment is the strongest predictor of essential genes [36].

However, the machine learning method cannot be used universally because of the lack of available experimental data in most genomes. Thus, a black box gene essentiality prediction algorithm, independent of experimental data, has been developed, which incorporates information on the biased gene strand distribution, the homologous search and the codon adaptation index (CAI) [37]. The algorithm achieved an AUC score of 0.81 when applied to the *Mycoplasma pulmonis* genome. It also achieved an accuracy of 78.9% and 78.1% in predicting essential genes in *Staphylococcus aureus* and *Bacillus subtilis* genomes, respectively.

Essential genes should be persistent during the long-term evolution [2]. Based on this idea, we developed a universal tool to offer gene essentiality annotations only via evolutionary information. Therefore, we apply phylogeny weighted orthology variable to reflect evolutionary information in searching essential genes. In this work, we used a workflow similar with that developed by [37] given that its outstanding performance. A gene is considered essential if its essential orthologs are persistent, especially in similar species. For estimating orthology, we used the reciprocal best hit (RBH) method, which was widely and effectively applied to map orthologs [38], [39], [40], [41]. The distance of phylogeny between species was computed using the Composition Vector (CV) method [42]. The tool is called as ***g***ene ***e***ssentiality ***p***rediction ***t***ool based on ***o***rthology and ***p***hylogeny (Geptop). The web server and open source standalone package implementing our method are freely available at http://cefg.uestc.edu.cn/geptop/.

## Materials and Methods

### Data sets

Database of essential genes (DEG) hosts essential genes identified by experimental techniques across a wide range of organisms [43]. The current version (6.8) contains 19 bacterial strains and 8 eukaryotes. The annotations of gene essentiality were obtained from the DEG database and the complete coding sequences of all 19 bacteria were taken from GenBank. We then classified genes into the essential set and the non-essential set according to gene annotations in GenBank for each genome. As a result, 19 essential sets and their corresponding non-essential sets were obtained.

### Searching orthologs and estimating phylogeny distance

The orthologous gene pairs between each pair of genomes were identified based on the reciprocal best hit (RBH) method. For two given genomes, one genome was used as the query and the other as the subject. A query-subject gene pair was confirmed if it was found that a gene in the query matches another gene in the subject by all-against-all Blastp search with a default E-value cutoff of 10. If there were multiple hits with the E values lower than the cutoff for a given query gene, the hit with the lowest E-value was masked as the best hit. Then, the query and subject was switched to confirm the subject-query gene pairs using the same procedure. The symmetrical hits between query-subject gene pairs and subject-query gene pairs were identified as orthologous gene pairs.

The composition vector (CV) method is used to estimate evolutionary distance [42]. To calculate the CV distance between two species, we first collected amino acid sequence data. Second, we computed the frequencies of six-peptides. Thirdly, a composition vector of dimension 20^6^ was obtained for each species by putting the ‘normalized’ frequencies in a fixed order. Fourthly, the correlation *C* between two species was determined by the cosine function of the angle between the two normalized vectors. Finally, the normalized distance *D* between them is defined to be: 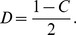



### Training workflow

Our method was based on phylogeny weighted orthology to predict the gene essentiality. To determine the optimal cutoff *S_0_* of identifying essential genes, we used *E. coli* as the test set, and the other 18 proteomes were used as the training set. The homology mappings were performed by RBH between *E. coli* and each of the proteomes. We identified the mapping score (*M*) as 1 if an *E. coli* gene was homologous and essential in the multiple genomes set during the homology mapping procedure. Meanwhile, the CV distance (*D*) between *E. coli* and each proteome was also computed. After mapping all 18 genomes, we defined the gene essentiality score *S_i_* for *i^th^ E. coli* gene: 
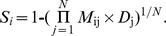
where *j* denotes the *j^th^* proteome in the multiple genomes set, *N* denotes the count of proteome and the range of *S* was between 0 and 1. In this training procedure, *N* equals 18. Finally, we looked for the optimal cutoff, *S_0_*, using a greed search method. If *S*>*S_0_*, the gene is predicted to be essential, otherwise, the gene is predicted to be non-essential.

### Performance assessment of method

The following parameters were measured in this study to assess the performance of the predictor: 
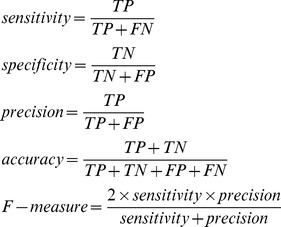
where *TP*, *FN*, *FP* and *TN* denote the true positives, false negatives, false positives and true negatives, respectively. The sensitivity parameter measures the proportion of essential genes that have been correctly identified. The specificity parameter represents the proportion of negatives that have been correctly predicted. The precision parameter is the probability that the essential genes were predicted as essential. The accuracy is the proportion of overall samples that have been correctly identified. The F-measure represents the harmonic mean of precision and sensitivity.

### Gene phyletic ages

We used the method described in [44], [45] to determine the phyletic ages for the genes in *E. coli*. For mapping orthologs, we applied RBH with E-value >10. We randomly collected 43, 68, 71, 75, 143 and 40 genomes from Archaea, Bacteria, Proteobacteria, Gammaproteobacteria, Enterobacteriaceae and *E. coli*, respectively. The number of hits required to assign a protein to the given age class was determined as half of the effective number of genomes. Consequently, the genome of *E. coli* was divided into six broad taxonomic classes. The unassigned genes were classified as strain-specific class.

## Results

### Homology mapping of essential *E. coli* genes to other organisms

We used a RBH method to search for orthologs of essential *E. coli* genes in 18 bacterial species. All bacteria had well-characterized essential genes determined by experimental techniques, and the annotations were extracted from DEG (Table 1). The evolutionary distance between each of the 18 bacterial genomes and *E. coli* was calculated with the CV method. The numbers of shared essential genes among *E. coli* and other genomes were correlated to their evolutionary distances (Spearman's *r* = −0.576, p<0.01). As an example, *Salmonella typhimurium* Ty2 is phylogenetically related to *E. coli*, sharing 283 *E. coli* essential genes (*Ecol*), of which 256 also show essentiality in *S. typhimurium* Ty2. *B. subtilis* shares 221 *Ecol* genes, of which 149 are consistent with *B. subtilis* essential genes (*Bsub*). With respect to the more distinct genomes such as *Mycoplasma* only share about 120 essential genes when compared with the *Ecol* dataset. Therefore the evolutionary distance between genomes does indeed have a significant impact on the outcome of homology mapping. Moreover, essential genes are not always conserved in similar species. There are 256 essential genes in *S. typhimurium* Ty2 closely related to essential *E. coli* genes, but only 84 *Ecol* orthologs also perform essential functions in the closely related *S. typhimurium* LT2. It is confusing when choosing the reference organism for homology mapping by essential function because essential genes do not always transfer across organisms regardless of the evolutionary distance. That is why we performed homology mapping using multiple genomes rather than a single genome.

**Table 1:** Detailed information regarding the 19 bacterial species investigated.

Organism	Abbreviation	Number of genes		Phylum
		Essential	Total	
*Acinetobacter baylyi*ADP1	*Abay*	499	3307	*Proteobacteria*
*Bacillus subtilis* 168	*Bsub*	271	4176	*Firmicutes*
*Caulobacter crescentus* NA1000	*Ccre*	480	3878	*Proteobacteria*
*Escherichia coli* MG1655	*Ecol*	296	4146	*Proteobacteria*
*Francisella novicida* U112	*Fnov*	390	1719	*Proteobacteria*
*Haemophilus influenzae* Rd KW20	*Hinf*	642	1657	*Proteobacteria*
*Helicobacter pylori* 26695	*Hpyl*	322	1573	*Proteobacteria*
*Mycobacterium tuberculosis* H37Rv	*Mtub*	614	4003	*Actinobacteria*
*Mycoplasma genitalium* G37	*Mgen*	378	475	*Tenericutes*
*Mycoplasma pulmonis*UAB CTIP	*Mpul*	310	782	*Tenericutes*
*Pseudomonas aeruginosa* UCBPP-PA14	*Paer*	335	5892	*Proteobacteria*
*Salmonella typhi* Ty2	*StypT*	352	4313	*Proteobacteria*
*Salmonella typhimurium* LT2	*StypL*	230	4423	*Proteobacteria*
*Staphylococcus aureus* N315	*SaurN*	302	2583	*Firmicutes*
*Staphylococcus aureus* NCTC 8325	*SaurC*	351	2891	*Firmicutes*
*Streptococcus pneumonia* TIGR4	*SpneT*	111	2105	*Firmicutes*
*Streptococcus pneumonia* R6	*SpneR*	133	2042	*Firmicutes*
*Streptococcus sanguinis* SK36	*Ssan*	218	2270	*Firmicutes*
*Vibrio cholerae* N16961	*Vcho*	779	3834	*Proteobacteria*

### Classifier training on the 18 bacterial genomes and cross-organism validation in *E. coli*

**Figure 1:**
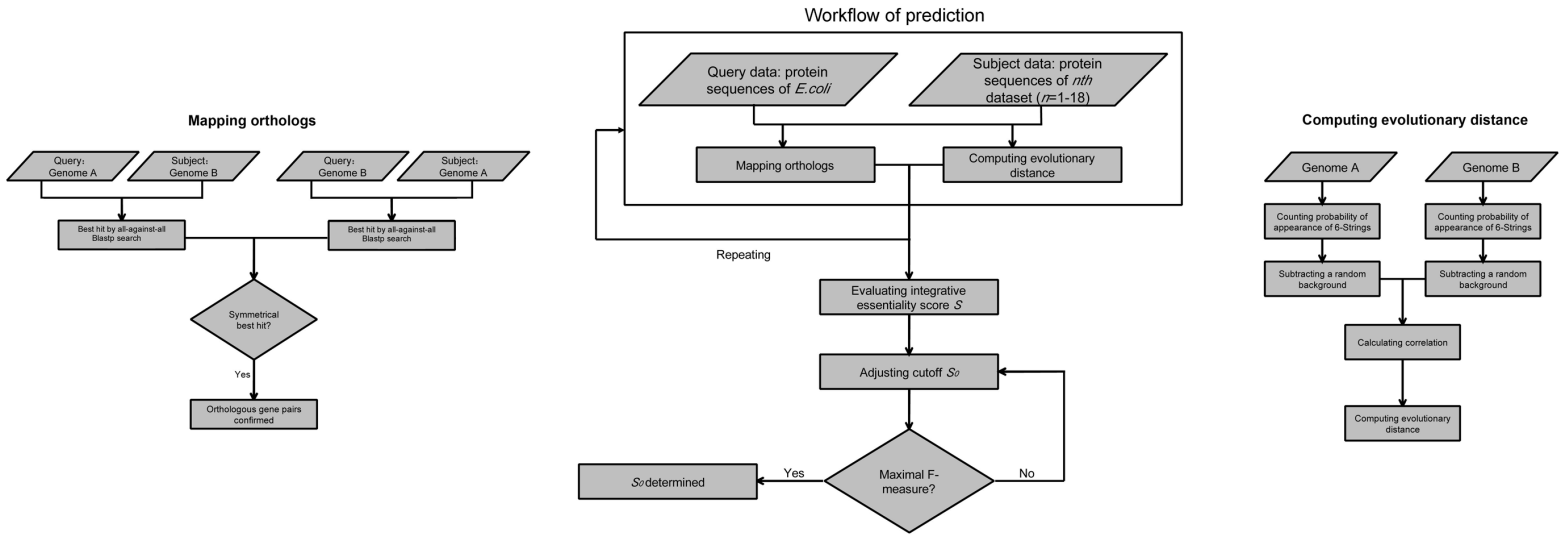
Training workflow based on 18 groups of essential genes and using *Ecol* as test.

**Figure 2:**
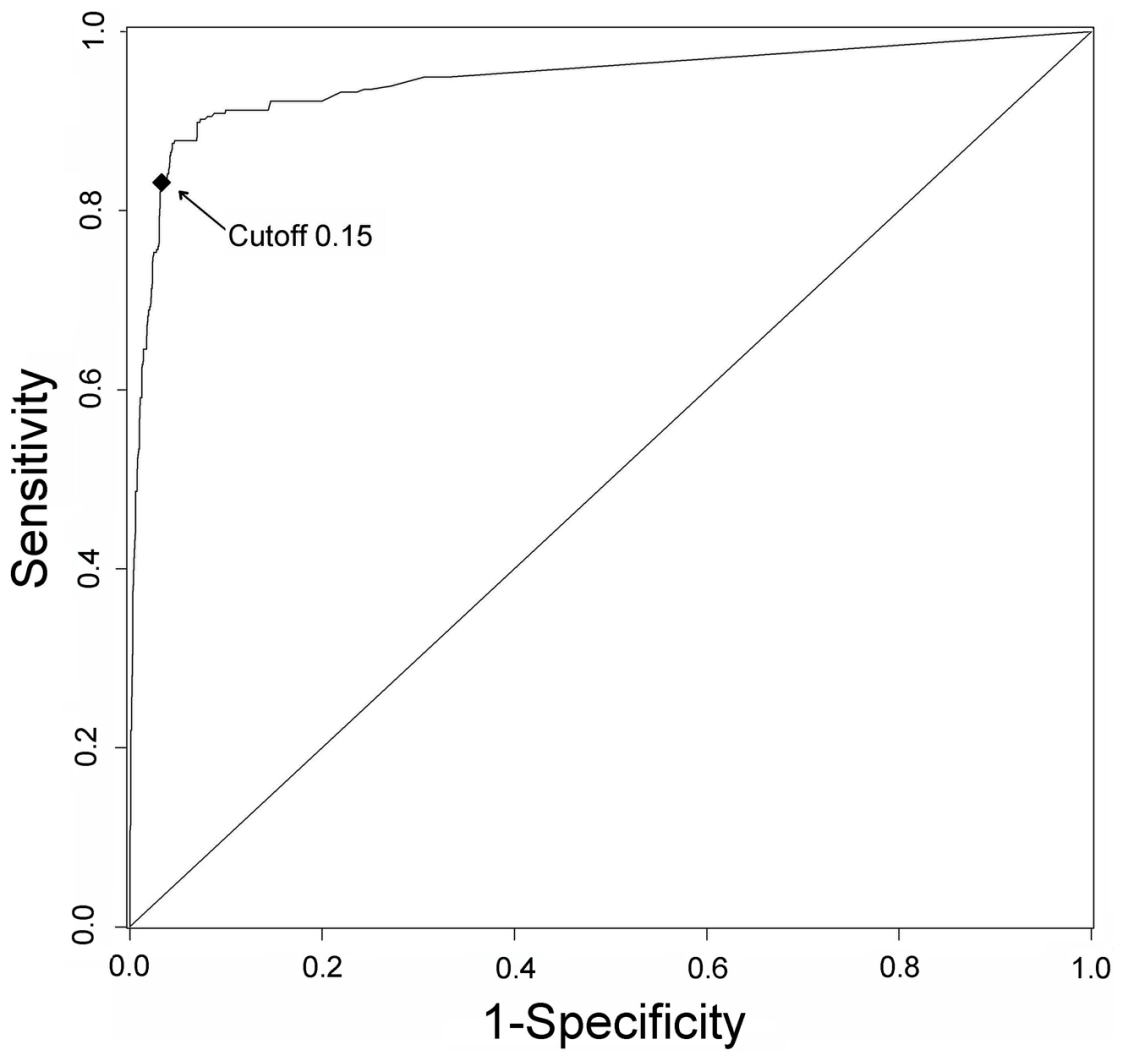
ROC curve of cross-organism validating for *Ecol*. Black diamond denotes the default cutoff of 0.15.

**Figure 3:**
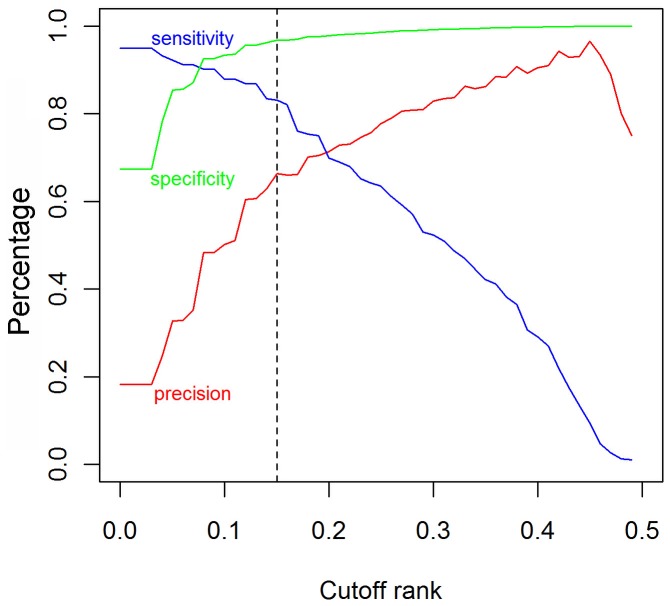
The precision, sensitivity and specificity in relation to the cutoff rank. The vertical dashed line represents the default cutoff of 0.15.

To determine the cutoff of identifying essential genes, we used 18 proteomes as searchable databank and then *E. coli* was applied to test; a detailed workflow is shown in Figure 1. Validation test of the classifier yielded an AUC score of 0.947 using *Ecol* (Figure 2). The changes in precision, sensitivity and specificity along with that of predicted essentiality score cutoff are illustrated in Figure 3. The number of non-essential genes was almost eight times greater than that of essential genes. To avoid the bias caused by excessive number of false essential genes and insufficient number of true essential genes, we used the harmonic mean of precision and sensitivity (F-measure) to determinate the threshold of classifying essential genes. This indicator is widely used for assessing the performance of various kinds of classifiers [46], [47], [48]. The classifier identified 371 essential genes in *E. coli* at (*S_0_* = 0.15) with the maximal F-measure. Of these predicted essential genes, 246 were true positives; however, 50 real essential genes were lost. Therefore, we were able to achieve a sensitivity of 83.1% and specificity of 96.8%.

The other broadly used essential dataset (*EcolP*) was downloaded from the PEC database [49]. These essential genes were also used as a validation set and the classifier yielded an AUC score of 0.978. This score is much better than the result of a recent study using the integrative machine learning systems [36] (AUC = 0.82–0.89). With the maximal F-measure, the classifier predicted 261 essential genes in *EcolP* (*S_0_* = 0.15) and achieved an accuracy of 96.7%. Because the cutoff of *S* determined by*EcolP* is as the same as by *Ecol*, we therefore determined *S_0_* to be 0.15.

**Figure 4:**
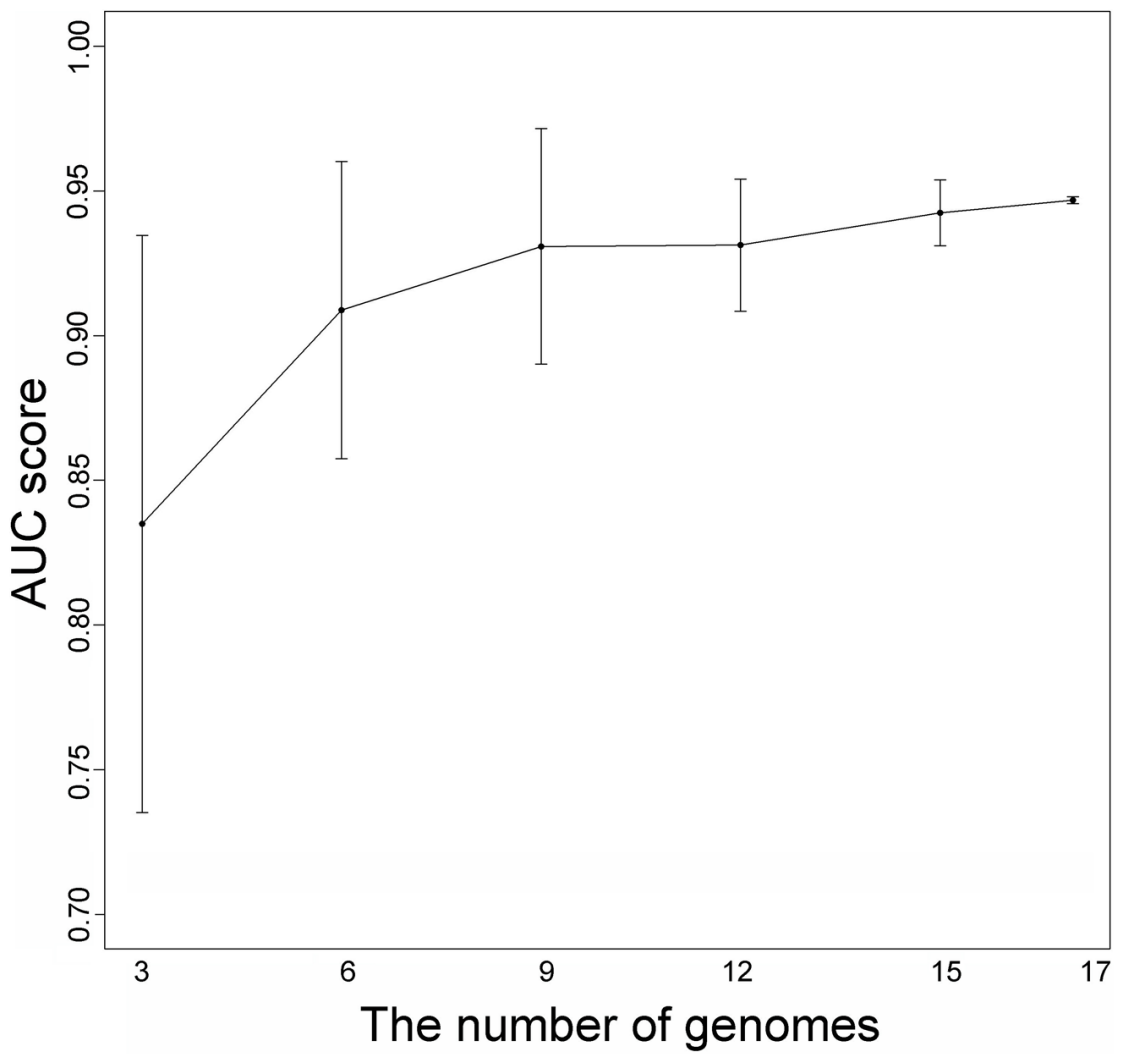
AUC scores of jackknife test. We randomly picked 10 times of 3, 6, 9, 12, 15, 17 genomes from the remaining 18 genomes and computed the AUC scores of predicting *Ecol*. Error bars are representing 90% confidence intervals on the estimates of the means.

For evaluating the effect of using different multiple genomes, we randomly picked 3, 6, 9, 12, 15 and 17 genomes, for 10 times of each case, from the remaining 18 genomes and computed the AUC scores of predicting *Ecol*. As can be seen from Figure 4, the application of more genomes indeed made the results of prediction better and more stable. Thus, the application of multiple genomes in the classifier can improve the power of prediction.

### Cross-validations of classifier

The proteins of the 19 collected organisms were randomly upset and then partitioned into ten samples. Of the ten newly generated proteomes, one proteome was used as the test set, and the remaining proteomes were used as the training set. We repeated this ten times, thus each of the ten proteomes was used once as the validation data. This ten-fold cross-validations in the random samples yielded an AUC score of 0.918. The results balanced between precision (0.512) and sensitivity (0.581) with the *S_0_* set at 0.05–0.07 and yielded an accuracy of 0.916.

**Figure 5:**
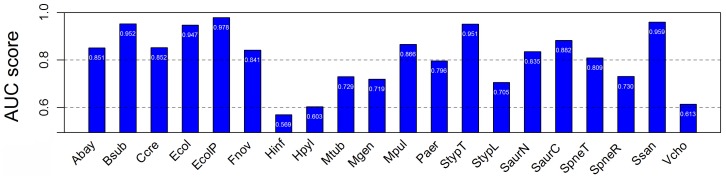
AUC scores from cross-organism Geptop prediction. The range of AUC is from 0.5 to 1.

Considering that the distinct evolutionary information was discarded during the random sampling, cross-organism validation was adopted to re-estimate the classifier. One of the 19 collected organisms was used as the test set, and the other ones were trained. Validation was repeated 18 times with each of the organisms being used exactly once as the validation data (For *Ecol* dataset, we have already performed this validation test above). Cross-organism predictions yielded AUC scores between 0.569 and 0.959 (Figure 5).

For overall predictions, 52.6% of their AUC scores were greater than 0.8, which indicated that classifier was significant and efficient. For example, the model Gram-positive bacterium *Bsub* yielded a high AUC score of 0.952. From all predictions, five yielded acceptable AUC scores between 0.7 and 0.8, while the other three predictions tended to be randomized. We applied the cutoff of 0.15 on the classifier to predict essential genes in 18 organisms and 15 of them exceeded an accuracy of 0.8 (Table 2).

**Table 2:** Cross-organism test accuracies of the Geptop.

Dataset	Precision	Sensitivity	Specificity	Accuracy
*Abay*	0.810	0.511	0.979	0.908
*Bsub*	0.665	0.753	0.974	0.959
*Ccre*	0.824	0.527	0.984	0.928
*Ecol*	0.663	0.831	0.968	0.958
*EcolP*	0.704	0.909	0.971	0.967
*Fnov*	0.787	0.633	0.950	0.878
*Hinf*	0.550	0.268	0.861	0.631
*Hpyl*	0.343	0.308	0.848	0.737
*Mtub*	0.686	0.316	0.974	0.873
*Mgen*	0.941	0.460	0.887	0.547
*Mpul*	0.914	0.581	0.964	0.812
*Paer*	0.503	0.496	0.971	0.944
*StypT*	0.734	0.793	0.975	0.960
*StypL*	0.254	0.444	0.929	0.903
*SaurN*	0.488	0.613	0.915	0.880
*SaurC*	0.620	0.650	0.945	0.909
*SpneT*	0.199	0.532	0.881	0.862
*SpneR*	0.232	0.541	0.875	0.854
*Ssan*	0.639	0.844	0.949	0.939
*Vcho*	0.675	0.285	0.965	0.827

### Geptop

Taking all the 19 bacteria as the multiple genomes set and adopting the same model and the threshold, we formed an online and also a standalone tool designated as Geptop. The Geptop web server first provided an online platform to detect essential gene sets across bacterial species using our classifier (http://cefg.uestc.edu.cn/geptop/). After a user submits the whole-proteome for a bacterial species in the FASTA format, the web server will automatically compute the essentiality score for each gene by comparing the orthology and phylogeny information for the 19 genome datasets (from DEG). The default cutoff is set at 0.15. The result will be automatically sent to the user via e-mail when the prediction is completed. Alternatively, a standalone version Geptop is also available. This package is dependent on Python, Biopython and BLAST+.

### Application of Geptop: predicting essential genes in sequenced bacterial genomes

**Figure 6:**
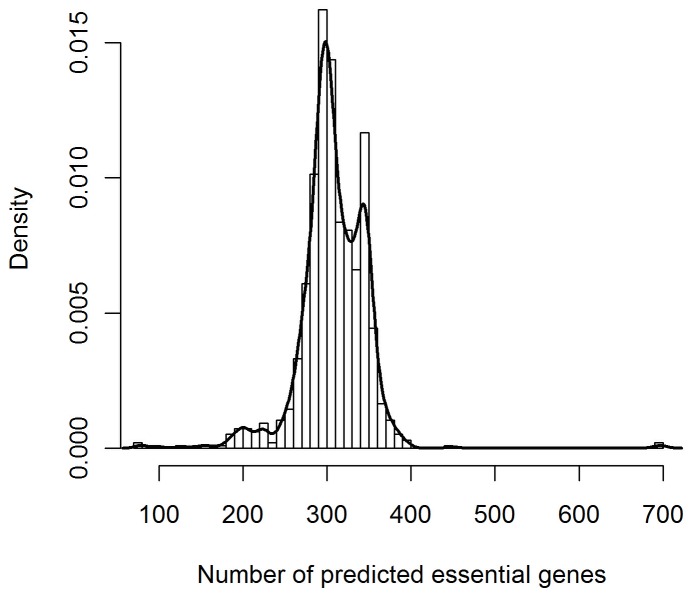
The distribution of predicted essential gene numbers.

We predicted essential genes in 968 sequenced bacteria, which are from 26 different phyla, using our Geptop method. Our webserver provides the details of these predictions at http://cefg.uestc.edu.cn/geptop/list.html. With the default cutoff (S = 0.15), most predictions identified 250–350 essential genes (Figure 6). Previously, the minimal gene set of cellular life has estimated approximately 250 candidates [3], [21]. Our result provides a piece of evidence for supporting this estimation.

A newly experimentally identified essential gene dataset from *Porphyromonas gingivalis* was also collected [50], which could be used to assess the performance of Geptop since this species belongs to a different phylum comparing the bacteria included in Geptop. As expected, 281 essential genes were predicted by our websever (Table S1 in File S1), 80% of them are experimentally identified. This prediction yields an AUC score of 0.77. We thus confirm that the method of Geptop does predict essential genes effectively even when applied in distantly organisms and most estimations could yield an AUC score exceed 0.70.

Since our method aims to find essential genes by evolutionary information, it might lose the species-specific essential genes. However, the species-specific genes (young genes) were found less likely essential than older genes [45]. As mentioned above, we lost 50 real essential genes for *Ecol* by the cross-organism prediction. We investigated the phyletic ages of these 50 genes and found 12 of them belong to strain-specific or *E. coli*-specific. That is to say, only 4% (12/296) essential genes were mistakenly classified because they are species-specific. Therefore, the performance of Geptop is acceptable if we do not specially focus on those species-specific essential genes.

## Discussions

### Geptop advantages

Cross-organism predictions yielded AUC scores between 0.69 and 0.89 using another integrative genomics method [36]. *Acinetobacter baylyi*, *P. aeruginosa* and *B. subtilis* was used to predict *EcolP*, and the AUC scores were between 0.82 and 0.89. When using *EcolP* to predict essential genes in *A. baylyi* (*Abay*) and *B. subtilis* (*Bsub*), the AUC scores were 0.80. The authors [36] did not use *EcolP* to carry out prediction for the essential set of *Paer* for the strain UCBPP-PA14, which is involved in our work. The study used only one genome for cross-validation. As mentioned in [36], RBH did not work well using only one genome. However, our tool consider putting in the phylogeny information (CV), could effectively work dependent of multiple genomes. We here used three of *EcolP*, *Bsub*, *Abay* and *Paer* for training, and the rest one for testing. The AUC scores of cross-organism predictions of *EcolP* and *Bsub* by Geptop are 0.91 and 0.85, respectively, which are slightly better when comparing with the integrative method. However, the AUC of *Abay* (0.77) shows a little weaker. When using 18 genomes for training, the power of method is improved, even *Abay* (AUC = 0.85) is better than the integrative method (Figure 5). Another machine learning system based on the integrative features was trained to identify *Ecol* using *Pseudomonas aeruginosa*, and to identify essential genes in *P. aeruginosa* (*Paer*) using *E. coli* [35]. It yielded an AUC of 0.81 and 0.80 in predicting *Ecol* and *Paer*, respectively. We also used *Bsub*, *Abay* and *Paer* to predict *Ecol* by Geptop then yielded an AUC of 0.88, simultaneously, adopted *Bsub*, *Abay* and *Ecol* to predict *Paer* then yielded an AUC of 0.77. Generally, our method is competitive with the integrative method when using only three genomes as training set.

**Figure 7:**
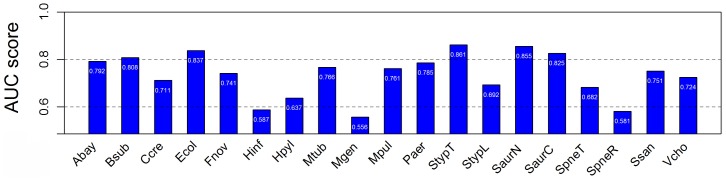
AUC scores from cross-organism tests using the integrative compositional information predictor. The range of AUC is from 0.5 to 1.

Although integrative genomics predictors scored highly in predicting bacterial essential genes, these classifiers usually rely on several biological features. The lack of functional experimental resources blocked the application of those algorithms. We investigated a different integrative genomics predictor based only on sequence compositional information, with 158 features considered. These features were amino acid usage (20 features), codon usage (64 features), codon position-specific nucleotide usage (12 features), 2-tuple codon position-specific nucleotide usage (48 features), and 14 features from CodonW (http://codonw.sourceforge.net). Details are listed in Table S2 (File S1). Genes were classified as essential or non-essential as given by the DEG annotations. We then used CD-HIT (http://www.bioinformatics.org/cd-hit/) to remove the redundant data. Because of the asymmetric numbers between the essential and non-essential sets, the negative sample set was randomly chosen from the non-essential set to have evenly sized groups. The six prediction models were obtained after the support vector machine training for the six groups of samples. The average prediction score of models for each bacterial genome was computed and this prediction yielded an AUC score (Figure 7). The Geptop predictor improved AUC scores when compared with this compositional bias based classifier by focusing on higher scoring groups that had an AUC higher than 0.8 yielded by the Geptop. However, most low AUC scoring bacteria showed a limited change. For example, the Geptop yielded a maximum AUC score of 0.959 when predicting the *Streptococcus sanguinis* essential set, which was significantly higher than the integrative compositional information predictor (AUC score of 0.751). For the lowest Geptop AUC score of 0.569 in *Haemophilus influenzae*, the compositional classifier yielded a similar score of 0.587.

The Geptop is partly based on homology mapping. Similarly, a recently study has also performed Blast searching as a feature of the prediction [37]. This algorithm integrated the homology searching (one-way alignment), the codon adaptation index and the biased distribution of essential genes in leading and lagging strands. This study provided accuracy of about 80% when combined using the essential dataset in the *Mycoplasma genitalium* (*Mgen*) and the essential genes for *Mycoplasma pulmonis* (*Mpul*) to predict essentiality in genomes of *B. subtilis* and *S. aureus*. In our study, we improved the performance of predicting *B. subtilis* (accuracy  = 92%) and *S. aureus* (accuracy  = 87%) by equally using *Mgen* and *Mpul*.

The T-iDT finds essential genes by comparing a bacterial gene set against the DEG [51]. This tool matches potential essential genes in the DEG database using Blastp with an E-value cutoff of 10^−10^ and bit score greater than 100. The tool is based only on homology mapping to search for essential sets, which differs in two aspects when compared with the Geptop. First, we performed homology mapping by RBH to search for optimally matched genes in each bacterial genome; second, evolutionary distance was considered when searching orthologs among distinct genomes. Additionally, we also identified the essential genes in 19 bacterial species using cross-organism T-iDT predictions. Because the aim of the T-iDT is to identify drug targets, the essentiality prediction results showed high average specificity of 0.759 and low average sensitivity of 0.316 (Table S3 in File S1). However, our cross-organism Geptop prediction improved both the average specificity of 17.8% and sensitivity of 23.1% by at the default cutoff of 0.15 compared with the T-iDT. On the other hand, Holman et al. [17] defined a Multiple Hit Score (MHS) to predict essential genes based on the top alignments to essential genes for each bacterial strain in the DEG. Their cross-organism test showed scaled AUC scores between 0.137 and 0.742 among 14 strains. *B. subtilis* matched the highest score, which was still significantly lower than that predicted by the Geptop (scaled AUC score of 0.904). Our method adopted a different discriminatory function with better results.

Generally, the effectiveness of our method is similar to those method integrating functional genomics data and sequence-related features when only adopting three genomes to train the model. Moreover, with the expansion of training genomes, we can obtain better prediction. On the other hand, although using the multiple genomes, the power of one-way alignment is much weaker than that of combined evolutionary features (RBH/CV) in the field of predicting essential genes. Since our prediction only use protein sequence, we believe that the Geptop could be widely applied in most bacteria rather than only in specific species.

### Features of predicted essential genes

Gene essentiality always correlates to codon bias [31]. Therefore, we investigated the difference of codon usage between essential genes predicted by Geptop across organisms and those identified by experimental techniques. The CAI is a species-dependent codon bias measure, and has been widely used as an empirical measure for gene expression, particularly in microbial genomes [52]. With this methodology, the collection of several ribosomal protein genes are chosen as a reference set of highly expressed genes for each genome. All genes for a given genome were classified into four groups: essential genes uniquely identified by experimental disruptions (experimental group); essential genes uniquely predicted by the Geptop (Geptop group); common essential genes; and common non-essential genes. As shown in Table 3, codon bias of the Geptop group was greater than that of the experimental group in 11 of 19 genomes (Mann-Whitney test, p<0.05). Seven genomes had a similar codon bias between the two groups. Only in *Francisella novicida* does the experimental group show a greater codon bias.

**Table 3:** Comparison of features between the experimental and the Geptop groups.

	CAI			The percentage of leading genes			Connectivity		
	Experimental		Geptop	Experimental		Geptop	Experimental		Geptop
*Abay*	0.553±0.064	= a	0.565±0.093	56.1%	<<^b^	70.0%	54±49	<<^a^	99±50
*Bsub*	0.522±0.071	<<	0.544±0.076	83.6%	<<	97.1%	54±47	<<	111±59
*Ccre*	0.593±0.124	<<	0.634±0.099	66.1%	>>	55.6%	45±37	<<	103±53
*Ecol*	0.481±0.100	<<	0.589±0.129	60.0%	<<	72.8%	52±40	<<	114±54
*Fnov*	0.646±0.056	>>	0.633±0.047	65.0%	=	67.2%	27±26	<<	85±40
*Hinf*	0.520±0.064	<<	0.601±0.097	52.8%	<<	62.4%	36±38	<<	114±50
*Hpyl*	0.698±0.036	<<	0.709±0.024	57.4%	=	58.4%	35±30	<<	105±41
*Mtub*	0.619±0.067	=	0.624±0.069	64.8%	<<	86.5%	47±41	<<	85±49
*Mgen*	0.715±0.041	=	0.715±0.029	83.3%	>>	63.6%	25±23	<<	55±26
*Mpul*	0.679±0.048	=	0.679±0.050	66.9%	>>	58.8%	21±19	<<	40±25
*Paer*	0.634±0.099	<<	0.692±0.102	58.6%	<<	72.6%	28±32	<<	100±45
*StypT*	0.491±0.116	<<	0.532±0.112	61.6%	=	66.3%	46±34	<<	77±47
*StypL*	0.454±0.086	<<	0.551±0.119	64.8%	<<	70.7%	26±26	<<	89±46
*SaurN*	0.580±0.086	=	0.585±0.088	80.3%	<<	90.2%	44±41	<<	65±44
*SaurC*	0.579±0.094	=	0.586±0.083	82.1%	<<	87.2%	28±26	<<	68±45
*SpneT*	0.388±0.081	<<	0.506±0.170	84.6%	=	88.2%	48±39	<<	106±49
*SpneR*	0.375±0.073	<<	0.492±0.167	90.2%	=	89.9%	23±23	<<	85±41
*Ssan*	0.416±0.114	=	0.447±0.141	76.5%	<<	91.3%	27±18	<<	88±41
*Vcho*	0.418±0.079	<<	0.507±0.112	58.3%	<<	65.4%	22±33	<<	113±57

a “ = ” denotes significance of the Mann-Whitney test was within 5%; “>>” denotes that the experimental group was greater than the Geptop group using the Mann-Whitney test at a 5% level; “<<” denotes converse case.

b “ = ” denotes that the difference between two groups was within 5%; “>>” denotes that the difference at a significance level of 5% for the experimental group was greater than that for the Geptop group; “<<” denotes converse case.

Essential genes are asymmetrically distributed in leading and lagging strands [27], [28]. We extracted positions of replication origin and terminus for each bacterium from the DoriC database [53], and then genes were assigned to the two replication strand types. In 11 genomes the Geptop group shows more uneven strand distribution (the difference exceeds 5%). Only three genomes with leading strand bias in the experimental group were greater than 5% of the Geptop group. There are still five genomes showing the difference within the 5% level.

Connectivity refers to the number of directly-interacting partners of a protein in protein-protein links [29]. Essential genes are likely to link higher connectivity and to be hubs. Interaction data were obtained from the STRING9 database [54], with a default confidence score cutoff of 0.4. The comparison result illustrated the identical extreme preference of the Geptop essential genes (Mann-Whitney test, p = 10^−4^–10^−47^). Average numbers of the protein-protein interaction for the Geptop groups were almost two- to five-fold greater than the average connectivity in the experimental groups. We obtained a very low AUC score when predicting *Vibrio cholerae* by cross-organism Geptop, but the average connectivity of Geptop group was five-fold higher than experimental group. On the other hand, connectivity of the Geptop group was only twice of that of the other group for another high scoring set of *Ecol*.

**Figure 8:**
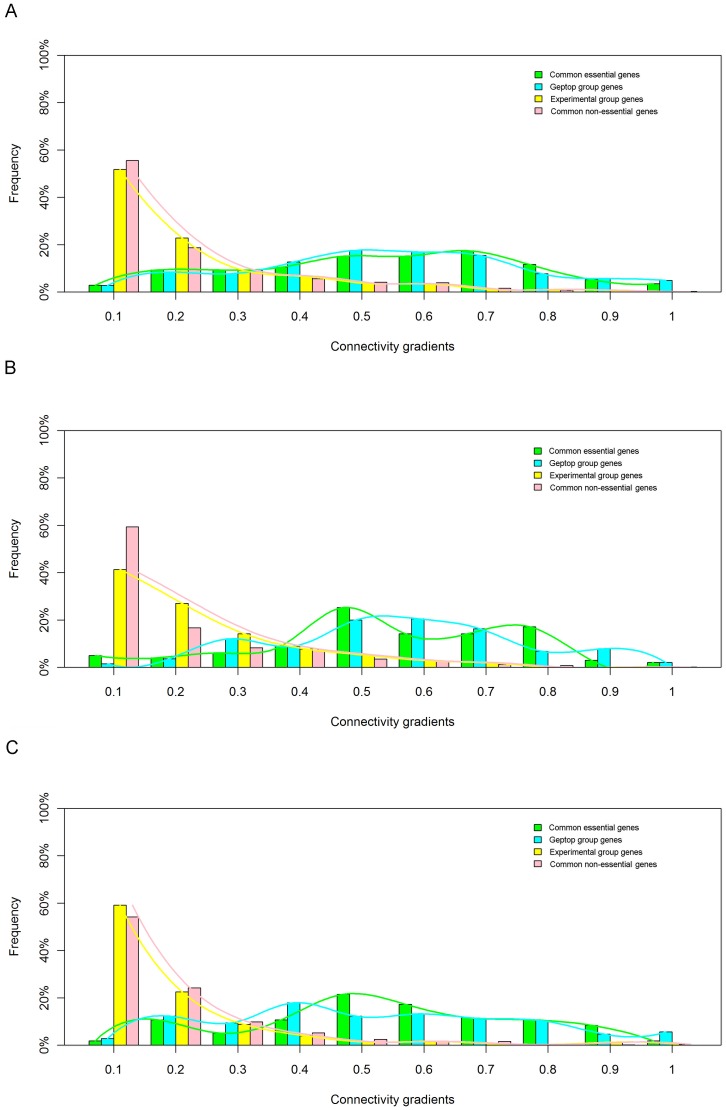
The connectivity distributions of four classes of genes in three bacteria with AUC lower than 0.7. (a) *H. influenza*; (b) *H. pylori*; (c) *V. cholera*.

We analyzed the correlation between AUC scores and the multiples of Geptop features over experimental features. Among the above three features, stepwise regression results showed that the AUC scores only related to the feature of connectivity difference (r = −0.533, p = 0.018). Therefore, if a prediction yields a lower AUC score it means that the numbers of protein-protein interactions for the Geptop groups could be significantly greater than the average connectivity of the experimental groups. There were three organisms, (*H. influenzae, Helicobacter pylori* and *V. cholerae*), that yielded very low AUC scores of 0.569–0.613. The three predictions lacked lots of essential genes identified by experimental techniques, however, these lacked genes show limited protein-protein interactions. As shown in Figure 8, the distribution of connectivity for the Geptop group was more similar to that for genes identified by both the Geptop and experimental techniques. However, the connectivity distribution of the experimental group was consistent with that of common non-essential genes.

Large-scale systematic experiments have provided important information about essential genes in many bacteria. Transposon mutagenesis technique, as the major experimental approach, is likely to mislabel short genes as essential, if insertions have been avoided by chance [9], [55]. The RNAi technique may not silence the expression of an essential gene entirely, but rather “knock down” its expression level [56]. The gene knockout method is the least error-prone approach for identifying essential genes; but this is expensive and time-consuming. The comparison of connectivity between the experimental and the Geptop groups suggested that genes uniquely predicted by the Geptop are more likely to be in protein-protein interaction network hubs, especially in the three organisms with low AUC scores. Moreover, another distinct classifier based on compositional bias also yielded low AUC scores (between 0.587 and 0.724) when predicting the three essential groups whose essential genes had been identified by transposon mutagenesis. In sum, we consider that the experimental identifications of essential genes for these bacteria were not quite accurate, or these genomes have so complex genomic architectures and evolutionary process that those simplistic features (compositional or evolutionary information) did not work well for predicting their essential genes.

In conclusion, our method yielded good AUC scores that are higher than integrative approaches and is expected to be applied widely in every species whose genome has been sequenced. Moreover, the essential genes predicted by the Geptop have more codon bias, distribution bias and abundant protein-protein interactions, which provide further evidence for the reliability of our method. With the availability of more reliable experimental essential sets potentially representing major phylogenetic lineages, the accuracy of our predicting method could be further improved.

## Supporting Information

File S1 Table S1, Geptop predicted essential genes in Porphyromonas gingivalis. Table S2, Features used in the integrative compositional information predictor. Table S3, Cross-organism test accuracy of the T-iDT.
(DOC)
