# Supplementary material for: Geptop: A Gene Essentiality Prediction Tool for Sequenced Bacterial Genomes Based on Orthology and Phylogeny
Source: PLoS One. 2013 Aug 15;8(8):e72343. doi: 10.1371/journal.pone.0072343 (PMC3744497; doi:10.1371/journal.pone.0072343)
Supplement: File S1 — Table S1, Geptop predicted essential genes in Porphyromonas gingivalis. Table S2, Features used in the integrative compositional information predictor. Table S3, Cross-organism test accuracy of the T-iDT. (DOC) [file pone.0072343.s001.doc]

**Table S1 Geptop predicted essential genes in *Porphyromonas gingivalis*.**

| GI number (essentiality score) | | | | | |
| --- | --- | --- | --- | --- | --- |
| 188994495(0.4640) | 188995734(0.4231) | 188995324(0.3792) | 188993888(0.3071) | 188993902(0.2542) | 188995335(0.1678) |
| 188995252(0.4640) | 188994492(0.4018) | 188995569(0.3792) | 188994386(0.3071) | 188995635(0.2542) | 188994143(0.1677) |
| 188995720(0.4640) | 188994494(0.4017) | 188995607(0.3792) | 188995326(0.3071) | 188995269(0.2541) | 188994240(0.1677) |
| 188995735(0.4640) | 188995067(0.4017) | 188995615(0.3792) | 188995925(0.3071) | 188995442(0.2541) | 188994437(0.1677) |
| 188994128(0.4440) | 188993865(0.4016) | 188995730(0.3792) | 188994258(0.3070) | 188995724(0.2541) | 188994628(0.1677) |
| 188994491(0.4440) | 188993906(0.4016) | 188995832(0.3792) | 188994693(0.3070) | 188994053(0.2266) | 188994733(0.1677) |
| 188995715(0.4440) | 188994013(0.4016) | 188995854(0.3792) | 188995070(0.3070) | 188995582(0.2266) | 188995273(0.1677) |
| 188993898(0.4439) | 188994145(0.4016) | 188994115(0.3562) | 188995316(0.3070) | 188995810(0.2266) | 188995490(0.1677) |
| 188994277(0.4439) | 188994219(0.4016) | 188995714(0.3562) | 188995438(0.3070) | 188994070(0.2265) | 188995655(0.1677) |
| 188994407(0.4439) | 188994224(0.4016) | 188995944(0.3562) | 188994780(0.3069) | 188994379(0.2265) | 188994267(0.1676) |
| 188994826(0.4439) | 188994257(0.4016) | 188994130(0.3561) | 188994104(0.2813) | 188994382(0.2265) | 188994351(0.1676) |
| 188994827(0.4439) | 188994381(0.4016) | 188994230(0.3561) | 188994364(0.2813) | 188994499(0.2265) | 188994476(0.1676) |
| 188995093(0.4439) | 188994678(0.4016) | 188994503(0.3561) | 188994376(0.2813) | 188994542(0.2265) | 188994498(0.1676) |
| 188995435(0.4439) | 188994758(0.4016) | 188994884(0.3561) | 188994408(0.2813) | 188994861(0.2265) | 188994533(0.1676) |
| 188995452(0.4439) | 188995021(0.4016) | 188995453(0.3561) | 188994511(0.2813) | 188995020(0.2265) | 188994730(0.1676) |
| 188995454(0.4439) | 188995245(0.4016) | 188995648(0.3561) | 188994705(0.2813) | 188995058(0.2265) | 188994968(0.1676) |
| 188995510(0.4439) | 188995322(0.4016) | 188995719(0.3561) | 188994876(0.2813) | 188995084(0.2265) | 188995356(0.1676) |
| 188995575(0.4439) | 188995451(0.4016) | 188995755(0.3561) | 188995015(0.2813) | 188995178(0.2265) | 188995446(0.1676) |
| 188995691(0.4439) | 188995669(0.4016) | 188993907(0.3560) | 188995441(0.2813) | 188995444(0.2265) | 188995842(0.1676) |
| 188995707(0.4439) | 188995706(0.4016) | 188994229(0.3560) | 188994163(0.2812) | 188994131(0.2264) | 188994059(0.1675) |
| 188995710(0.4439) | 188995711(0.4016) | 188994233(0.3560) | 188994173(0.2812) | 188994367(0.2264) | 188994132(0.1675) |
| 188995717(0.4439) | 188995718(0.4016) | 188994502(0.3560) | 188995017(0.2812) | 188994384(0.2264) | 188994250(0.1675) |
| 188995722(0.4439) | 188995732(0.4016) | 188994529(0.3560) | 188995440(0.2812) | 188994432(0.2264) | 188994505(0.1675) |
| 188995725(0.4439) | 188995845(0.4016) | 188994645(0.3560) | 188995859(0.2812) | 188994486(0.2264) | 188994623(0.1675) |
| 188995726(0.4439) | 188995924(0.4016) | 188995305(0.3560) | 188995882(0.2812) | 188994690(0.2264) | 188994634(0.1675) |
| 188995727(0.4439) | 188994001(0.4015) | 188995512(0.3560) | 188994839(0.2811) | 188994888(0.2264) | 188994695(0.1675) |
| 188995885(0.4439) | 188994677(0.4015) | 188995562(0.3560) | 188995082(0.2811) | 188995566(0.2264) | 188995332(0.1675) |
| 188995908(0.4439) | 188995439(0.4015) | 188995931(0.3560) | 188995932(0.2811) | 188995568(0.2264) | 188995334(0.1675) |
| 188995239(0.4233) | 188995511(0.4015) | 188994162(0.3321) | 188994052(0.2810) | 188995626(0.2263) | 188995819(0.1675) |
| 188994829(0.4232) | 188995733(0.4015) | 188994487(0.3321) | 188994500(0.2810) | 188995917(0.2263) | 188995869(0.1675) |
| 188995434(0.4232) | 188995923(0.4015) | 188994603(0.3321) | 188994630(0.2810) | 188994889(0.1977) | 188995801(0.1674) |
| 188995479(0.4232) | 188994489(0.3794) | 188994849(0.3321) | 188995019(0.2810) | 188995416(0.1977) |  |
| 188995697(0.4232) | 188994504(0.3794) | 188994994(0.3321) | 188995096(0.2810) | 188993961(0.1976) |  |
| 188995704(0.4232) | 188993899(0.3793) | 188995090(0.3321) | 188995870(0.2810) | 188994507(0.1976) |  |
| 188995705(0.4232) | 188994031(0.3793) | 188995457(0.3321) | 188995614(0.2545) | 188995346(0.1976) |  |
| 188995713(0.4232) | 188994297(0.3793) | 188994037(0.3320) | 188995747(0.2545) | 188995494(0.1976) |  |
| 188995716(0.4232) | 188994488(0.3793) | 188994534(0.3320) | 188994262(0.2544) | 188995567(0.1976) |  |
| 188995723(0.4232) | 188994558(0.3793) | 188994856(0.3320) | 188994284(0.2544) | 188995649(0.1976) |  |
| 188995728(0.4232) | 188994624(0.3793) | 188995108(0.3320) | 188994490(0.2544) | 188993897(0.1975) |  |
| 188995729(0.4232) | 188994851(0.3793) | 188995248(0.3320) | 188994531(0.2544) | 188994221(0.1975) |  |
| 188995736(0.4232) | 188995315(0.3793) | 188995311(0.3320) | 188994543(0.2544) | 188994222(0.1975) |  |
| 188994055(0.4231) | 188995708(0.3793) | 188995351(0.3320) | 188994633(0.2544) | 188994759(0.1975) |  |
| 188994142(0.4231) | 188994054(0.3792) | 188995437(0.3320) | 188994862(0.2544) | 188994995(0.1975) |  |
| 188994218(0.4231) | 188994510(0.3792) | 188995579(0.3320) | 188994952(0.2544) | 188995313(0.1975) |  |
| 188995242(0.4231) | 188994666(0.3792) | 188995650(0.3320) | 188995369(0.2544) | 188995621(0.1975) |  |
| 188995436(0.4231) | 188994683(0.3792) | 188995759(0.3320) | 188995881(0.2544) | 188993960(0.1974) |  |
| 188995696(0.4231) | 188994739(0.3792) | 188995709(0.3319) | 188995883(0.2544) | 188994099(0.1974) |  |
| 188995712(0.4231) | 188994828(0.3792) | 188994688(0.3072) | 188994139(0.2543) | 188994484(0.1974) |  |
| 188995721(0.4231) | 188995069(0.3792) | 188995807(0.3072) | 188995053(0.2543) | 188995580(0.1974) |  |
| 188995731(0.4231) | 188995310(0.3792) | 188995811(0.3072) | 188995553(0.2543) | 188994287(0.1973) |  |

**Table S2** Features used in the integrative compositional information predictor.

| **Feature** | **Description** |
| --- | --- |
| Amino acid usage | The frequency of 20 kinds of amino acids. |
| Codon usage | The frequency of 64 common codons. |
| Codon site specific nucleotide usage | The frequency of A, G, C and T in three codon positions. |
| 2-turple codon site specific nucleotide usage | The frequency of 2-turple nucleotide usage of two continuous codons in three codon position. For examples, the feature AT1st means that the frequency of both finding A in codon 1st positions and finding T in next codon 1st positions. |
| CodonW | Silent base compositions (G3s,T3s, C3s, A3s), Codon Adaptation Index, Codon Bias Index, Frequency of Optimal codons, The effective number of codons, G+C content, G+C content 3rd position of synonymous codons, Frequency of synonymous codons, Gene length (amino acids), Hydropathicity score and Aromaticity score. |

**Table S3 Cross-organism test accuracy of theT-iDT.**

| Dataset | Sensitivity | Specificity | Accuracy |
| --- | --- | --- | --- |
| *Abay* | 0.478 | 0.766 | 0.708 |
| *Bsub* | 0.316 | 0.673 | 0.600 |
| *Ccre* | 0.393 | 0.779 | 0.701 |
| *Ecol* | 0.349 | 0.573 | 0.528 |
| *Fnov* | 0.357 | 0.865 | 0.762 |
| *Hinf* | 0.439 | 0.861 | 0.775 |
| *Hpyl* | 0.151 | 0.879 | 0.736 |
| *Mtub* | 0.434 | 0.772 | 0.703 |
| *Mgen* | 0.280 | 0.991 | 0.846 |
| *Mpul* | 0.270 | 0.970 | 0.828 |
| *Paer* | 0.270 | 0.502 | 0.455 |
| *StypT* | 0.427 | 0.611 | 0.574 |
| *StypL* | 0.198 | 0.547 | 0.476 |
| *SaurN* | 0.329 | 0.780 | 0.688 |
| *SaurC* | 0.361 | 0.795 | 0.707 |
| *SpneT* | 0.117 | 0.786 | 0.650 |
| *SpneR* | 0.119 | 0.790 | 0.654 |
| *Ssan* | 0.262 | 0.809 | 0.698 |
| *Vcho* | 0.456 | 0.672 | 0.628 |
